# The diagnostic significance of circulating miRNAs and metabolite profiling in early prediction of breast cancer in Egyptian women

**DOI:** 10.1007/s00432-022-04492-2

**Published:** 2022-12-02

**Authors:** Safinaz E. El-Toukhy, Sherien M. El-Daly, Mahmoud M. Kamel, Heba K. Nabih

**Affiliations:** 1 Medical Biochemistry Department, Medicine and Clinical Studies Research Institute, National Research Centre, 33 El-Bohouth st., Dokki, P.O. 12622, Giza, Egypt; 2 Cancer Biology and Genetics Laboratory, Centre of Excellence for Advanced Sciences, National Research Centre, Giza, Egypt; 3 Laboratory Department, Baheya Hospital for Early Detection and Treatment of Breast Cancer, National Cancer Institute, Cairo University, Giza, Egypt

**Keywords:** HER2/neu, miR-145, miR-382, miR-21, Liquid chromatography/mass spectrometry, Glutamic acid

## Abstract

**Objective:**

Breast cancer (BC) is one of the most commonly diagnosed solid malignancies in women worldwide.

**Purpose:**

Finding new non-invasive circulating diagnostic biomarkers will facilitate the early prediction of BC and provide valuable insight into disease progression and response to therapy using a safe and more accessible approach available every inspection time. Therefore, our present study aimed to investigate expression patterns of potentially circulating biomarkers that can differentiate well between benign, malignant, and healthy subjects.

**Methods:**

To achieve our target, quantitative analyses were performed for some circulating biomarkers which have a role in the proliferation and tumor growth, as well as, glutamic acid, and human epidermal growth receptor 2 (HER2) in blood samples of BC patients in comparison to healthy controls using qRT-PCR, liquid chromatography/mass spectrometry (LC/MS/MS), and ELISA.

**Results:**

Our findings showed that the two miRNAs (miRNA-145, miRNA-382) were expressed at lower levels in BC sera than healthy control group, while miRNA-21 was expressed at higher levels in BC patients than control subjects. Area under ROC curves of BC samples revealed that AUC of miRNA-145, miRNA-382, miRNA-21, and glutamic acid was evaluated to equal 0.99, 1.00, 1.00 and 1.00, respectively. Besides, there was a significantly positive correlation between miRNA-145 and miRNA-382 (*r* = 0.737), and a highly significant positive correlation between miRNA-21 and glutamic acid (*r* = 0.385).

**Conclusion:**

Based on our results, we conclude that the detection of serum miRNA-145, -382 and -21 as a panel along with glutamic acid, and circulating HER2 concentrations could be useful as a non-invasive diagnostic profiling for early prediction of breast cancer in Egyptian patients. It can provide an insight into disease progression, discriminate between malignancy and healthy control, and overcome the use limitations (low sensitivity and specificity, repeated risky exposure, and high cost) of other detecting tools, including mammography, magnetic resonance imaging, and ultrasound.

**Supplementary Information:**

The online version contains supplementary material available at 10.1007/s00432-022-04492-2.

## Introduction

Breast cancer (BC) is the second most common cancer in women worldwide, and one of the leading cancer-causing deaths overall **(**Ibrahimet al. 2020). According to the World Health Organization (WHO) report in 2020, more than two million women were diagnosed with BC and about 685,000 deaths occur globally (Rakhmina et al. 2021; WHO 2020). BC can be classified as: benign, which is not considered cancerous, not life threatening, grows slowly, and does not invade or spread to other parts of the body; or malignant, which has the potential to be metastatic and life threatening (Wei et al. 2020). Therefore, to minimize the rate of BC death and improve the prognosis of outcome with better therapeutic options, early non-invasive detection of BC would be a promising approach that facilities the diagnosis and discrimination of BC for personalized medications (Li et al. 2020; Mar-Aguilar et al. 2013). Liquid biopsy represents a rich source of multiple circulating biomarkers that could be considered as non-invasive promising diagnostic factors, which discriminate between healthy, benign, and malignant candidates for early detection of breast cancer (Underwoodet al. 2020; Marrugo-Ramírez et al. 2018).

MicroRNAs (miRNAs) are a set of small sequence (18–25 nucleotides) non-coding RNAs that regulate gene expressions at the transcriptional or post-transcriptional level by sequence-specific binding to the 3′ untranslated regions (3′ UTR) of the target gene, leading to the degradation or translation inhibition of the gene (Lv et al. 2019). Thus, miRNAs have an important role in controlling many biological processes such as cell growth, proliferation, invasion, differentiation, adhesion, apoptosis, and cellular metabolic pathways (Orlandella et al. 2021; El-Daly et al., 2020; Nabih 2020; Pedroza-Torres et al. 2019; Yanwirasti et al.^ 2017^; Pinweha et al. 2016). Abnormal alterations of miRNAs expression patterns are frequently observed in various cancers, including BC. Because of their distinct expression pattern in cancer and noticeable stability in blood, miRNAs are considered to be highly promising biomarkers for BC early diagnosis (Rakhminaet al. 2021; Mar-Aguilar et al. 2013).

MiRNA-145 was reported to be located in a fragile region of chromosome 5 (5q32-33) and played a profound role in the inhibition of cell growth, proliferation, angiogenesis, invasion, and migration of human BC through targeting multiple genes such as epidermal growth factor receptor (EGFR), vascular endothelial growth factor (VEGF), human epidermal growth factor receptor (HER3), cmyc, hypoxia-inducible factor 2 alpha (HIF2a), sex-determining region Y-box2 (SOX2), rho-associated coiled-coil kinase (ROCK1), RTKN (rhotekin), octamer-binding transcription factor 4 (Oct4), and transforming growth factor-β1 (TGF-β1) (Ibrahim et al. 2020; Ye et al. 2019; Zeinali et al. 2019; Ding et al. 2017; Hu et al. 2012; Kim et al. 2011; Wang et al. 2009). Accordingly, the down-regulation of the tumor suppressor miRNA-145 contributes to the metastasis and progression of the tumor and is considered to be a potential biomarker for BC diagnosis, screening, and prognosis (Lv et al. 2020; XU et al. 2019; Gonzalez-Villasana et al. 2019; Quan et al. 2018).

MiRNA-382 locates on the 14q32 locus and its expression level in BC is critically associated with cell viability, proliferation, migration, invasion, and survival (Gonzalez-Villasanaet al. 2019; Feng et al. 2017; Tao and Wu 2016). The function of miRNA-382 was detected to be mediated via its counteracting genes, which have a role in the promotion of proliferation and metastasis in breast cancer cells (Zhang et al. 2019; Lv et al. 2019). Additionally, it could potentially be used as a reported non-invasive biomarker that can differentiate between BC patients and healthy candidates (Fu et al. 2016; Mar-Aguilar et al. 2013).

MiRNA-21oncogene is located on the chromosome 17q23.2 region which is frequently amplified in breast cancer. The up-regulation of miRNA-21 results in the promotion of cancer growth, proliferation, invasion, angiogenesis, and metastasis via targeting of many genes involved in apoptosis and tumor suppression, including programmed cell death protein 4 (PDCD4), tropomyosin 1 (TPM1), RAS p21 protein activator (RASA1), phosphatase tensin and homolog (PTEN), MASPIN, P53, B cell lymphoma 2 (Bcl2), signal transducer and activator of transcription 3 (STAT3), and leucine zipper transcription factor-like 1 (LZTFL1). Hence, miRNA-21 is a useful early diagnostic biomarker that discriminates between malignant breast cancer, benign breast cancer, and healthy control (Wu 2020; Savitri et al. 2020; Elzoghby et al. 2019; Wang et al. 2019; Zhang et al. 2016a, b; Gao et al. 2013; Yan et al. 2008).

Along with the enormous challenge of early diagnosis of BC, which is widely accredited as the key to successful therapy, mass spectrometry-based metabolomics is a promising approach for detecting the concentration of endogenous metabolic biomarkers produced by cancerous cells during their growth, such as alanine, aspartate, and glutamate that have a vital role in BC development (Yang et al. 2020). The accumulation of glutamic acid in the body might lead to the down-regulation of its derivative glutamine; consequently, this would promote the incidence of BC by activating mammary epithelial cells proliferation by enhancing the production of ATP and nucleotides biosynthesis (Pietkiewicz et al. 2021; El Ansari et al. 2018; Coloff et al. 2016). Additionally, elevated levels of glutamic acid via glutaminolysis could maintain the Krebs cycle, which is the main source of energy in the cells (Dowling et al. 2015). Up-regulated glutamic acid levels were suggested to distinguish between healthy, benign, and BC patients, which could be helpful as an early diagnostic biomarker in breast carcinoma (Wang et al. 2018).

It is well known that breast cancer cells expressing human epidermal growth factor receptor 2 (HER2) have a poor prognosis and survival associated with an increased rate of proliferation, invasion, angiogenesis, metastasis, and recurrence, as compared to HER2-negative tumors. HER2/neu is a proto-oncogene located on chromosome 17q21 and encodes for p185, which is a transmembrane glycoprotein with an intrinsic tyrosine kinase activity domain that regulates cellular growth and differentiation **(**Iqbal and Iqbal 2014; Ferretti et al. 2007). HER2 could be used as circulating diagnostic biomarkers with the potential to provide a valuable prediction of disease progression and response to therapy (Wu and Chu 2022).

Since the main aim of this research is to explore circulating biomarkers that would predict the onset of breast cancer, the expression levels of miRNAs-145, -382, and -21 panel were assessed using qRT-PCR, besides HER2/neu by ELISA, as well as metabolomics (amino acids profiling) by LC/MS/MS in the blood samples of healthy, benign, and breast cancer candidates.

## Subject and methods

### Participant selection criteria

In the current research study, serum samples from 80 women were collected. Among them were 30 newly diagnosed breast carcinoma patients (stage I, II, and III), 30 women with benign breast disease, and 20 age-matched healthy women as control. The mean age range of the enrolled cases was 39–62 years for early-diagnosed breast cancer cases, 27–58 years for benign breast patients, and 41–53 for healthy controls. Patients included in our study were recruited from October 2020 to February 2021from Baheya Hospital, Giza, Egypt, for early detection and treatment of breast cancer. Each subject’s diagnostic medical report, including data such as age, diagnosis (benign or early-stage breast cancer), type, grade, estrogen, and progesterone status (if cancer), was available. The breast cancer diagnosis was confirmed by physical, radiological, and histopathological examinations. All samples were collected before surgery or any chemotherapy and radiotherapy intervention. None of the included patients were suffering from any chronic disease, diabetes, hypertension, or any other type of cancer. Additionally, none of the healthy controls had a history of malignancy or chronic hepatitis, or liver cirrhosis and was verified to be healthy by physical examination. Exclusion criteria included those with late stage or metastatic cancer or had undergone modified radical mastectomy or breast-conserving surgery, chemotherapy, targeted therapy, adjuvant radiotherapy, or endocrine therapy. All patients had signed an informed consent to be enrolled in the study. This study was approved by the Bioethical Committee of the National Research Centre (Ethical Clearance Document Registry Number 19382).

### Methods

#### Blood samples

Blood samples (3 ml) were withdrawn and divided into two parts: 0.5 ml on EDTA for the preparation of dried blood spot (DBS) for liquid chromatography/mass spectrometry **(**LC/MS/MS) technique; and the rest on a gel vacutainer tube, centrifuged to separate serum for the assessment of tumor diagnostic biomarkers by qRT-PCR and ELISA assays. Blood samples were first centrifuged at 1600 rpm for 10 min at 4 °C for serum separation. A second centrifugation step for serum samples was conducted to remove residual platelets for efficient extraction of cell-free RNA.

#### RNA isolation and qRT-PCR quantification of microRNAs

As recommended by Link et al. (2019), all procedures related to RNA isolation or quantification was conducted under RNAse-free conditions. Isolation of cell-free RNA, including miRNAs, from serum samples, was performed using the miRNeasy Mini Kit (Qiagen, Hilden, Germany) following the protocol provided by the manufacturer. Following the extraction steps, total RNA including miRNAs was eluted from the miRNeasy column using 15 μl of RNase-free water. The quantity and purity of eluted RNA were assessed by NanoDrop™ One Microvolume UV–Vis Spectrophotometer (Thermo Scientific™) directly upon isolation. RNA was reverse transcribed using the miRCURY LNA Reverse Transcription Kit (Qiagen, Cat#339340) following the guidelines of the provided kit. The cDNA was kept at −20 °C till further processing by real-time PCR. Expression analysis of miRNAs was evaluated by real-time PCR using miRCURY LNA SYBR Green PCR Kit (Cat# 339346) and miRCURY LNA miRNA PCR assays for miR-21-5p, miR-145-5p, miR-328-5p, and miR-16-5p. The PCR cycling conditions were set as follows: PCR initial heat activation for 2 min at 95 °C, followed by two-step cycling of denaturation for 10 s at 95 °C, and a combined annealing/extension step at 60 s for 56 °C. A total of 40 cycles were performed, followed by a melting curve analysis. For each miRNA expression analysis, each sample was run in triplicate. The expression levels were normalized to miR-16, as suggested by several reports on the validity of miR-16 as an internal reference in circulating miRNA analysis **(**El-Daly et al. 2019; Lange et al. 2017; Witwer 2015). ∆Ct was calculated for each sample by normalizing the Ct value of the miRNA of interest to the Ct value of the miR-16 (normalizer). ∆∆Ct was then calculated by subtracting the ∆Ct of the test sample from the mean ∆Ct values of control samples. Data were presented as relative mRNA expression (fold change).

#### Analysis of L-amino acids by liquid chromatography/mass spectrometry (LC/MS/MS)

##### Sample preparation

As described by Wang et al. (2016), a 3 mm (diameter) disc was punched from each DBS paper. The discs were placed into the Millipore Multi Screen HV 96-well plate (Millipore, Billerica, MA, USA) for metabolite extraction. Briefly, for each well containing a DBS disc, 100 μl working solution was added. Then, the plates were centrifuged at 1500×*g* for 2 min after gentle shaking for 20 min. By using new flat-bottom 96-well plates, the filtrate was collected. For each plate, four randomly selected blank wells were added with two low-level and two high-level QC control solutions individually. Pure nitrogen gas was used to dry the QC and filtrate samples at 50 °C. Further, 60 μl of acetyl chloride/1-butanol mixture (10:90 v/v) was added to the dried samples at 65 °C for 20 min for derivatization. The derivatized samples were dried again with nitrogen gas at 50 °C. For metabolomic analysis, each dried sample was dissolved in 100 μl fresh mobile phase solution.

##### Metabolomic analysis

The direct injection MS metabolomic analysis was conducted by using an AB Sciex 4000 QTrap system (AB Sciex, Framingham, MA, USA). The equipped ion source was an electrospray ionization source. A 20 μl sample was injected for each run. The mobile phase was 80% acetonitrile aqueous solution. The initial flow rate was 0.2 ml/min. Afterward, the flow rate was set to 0.01 ml/min within 0.08 min, kept constant until 90 s, returned to 0.2 ml/min within 0.01 min, and held constant for another 30 s. The ion spray voltage was 4.5 kV. The curtain gas pressure was set at 20 psi. A 35 psi pressure was applied to the ion source gas 1 and gas 2. The auxiliary gas temperature was maintained at 350 °C. Analyst v1.6.0 software (AB Sciex) was used for system control and data collection. ChemoView 2.0.2 (AB Sciex) was used for data preprocessing. Partial least squares discriminant analysis (PLS-DA) was performed by using SIMCA-P v12.0 (Umetrics, Umeå, Sweden).

#### Quantification of HER2/neu by ELISA assay

Human HER2/neu was estimated by enzyme-linked immunosorbent assay (ELISA) in sera of all collected samples, according to the method of Shukla et al. (2016) with some modifications. The ELISA kit (sunLong Biotech Co., China) uses the sandwich ELISA technique as the principle of the working methodology. The 96-well ELISA strip plate provided in the kit had been pre-coated with a primary antibody specific to HER2. Standards or samples were added to the appropriate wells and combined with the specific antibody. Then, horseradish peroxidase (HRP)-conjugated secondary antibody specific for the primary HER2 was added to each well and incubated. Free unconjugated components were washed away through repeated washing steps. Finally, 3,3′,5,5′- tetramethylbenzidine (TMB) substrate solution was added to each well. Only those wells that contain the detected antigen and the corresponding HRP-conjugated antibody would appear blue in color and then turn yellow after the addition of the stop solution. The optical density (OD) was measured by microplate ELISA reader at a wavelength of 450 nm (Tristartlb 942 microplate reader; Berthold, Germany). The OD values are proportional to the concentration of the detected antigen. The concentration of HER2/neu in each sample was calculated from a standard curve.

#### Statistical analysis

All data were represented as mean ± standard error of the mean (SEM) with statistically significant consideration at *P* ≤ 0.05 (*), *P* ≤ 0.01 (**), *P* ≤ 0.001 (***), and **** *P* < 0.0001 using a two-sided independent Student's *t* test. All experiments were repeated in triplicate. The potential of biomarkers and their diagnostic efficacies were evaluated by receiver operating characteristic (ROC) analysis. Moreover, correlations between estimated biomarkers were analyzed using the Pearson correlation coefficient (*r*). The statistical analyses and calculations were performed using Statistical Package for the Social Sciences (SPSS, Inc., Chicago USA) version 16.0 software. Graph Pad Prism 8.0.1 (Graph Pad Software Inc., USA) was used for the figures’ output, correlation coefficient, and ROC analysis.

## Results

### Clinicopathological features and medical recorded data for the enrolled breast cancer patients

According to the diagnostic medical sheet of each included breast carcinoma patient (*n* = 30), the type of malignancy was differentiated as follows: 83.33% (25/30) of cases were diagnosed as invasive ductal carcinoma (IDC), 10% (3/30) of patients were characterized as ductal carcinoma in situ (DCIS), and 6.66% of patients (2/30) were diagnosed with multifocal carcinoma. The grade of tumor was distributed among patients as 13.33% with grade I (1/30), 70% with grade II (21/30), and 26.66% with grade III (8/30). Concerning endocrine receptor expression in enrolled cases, there were 76.66% (23/30) and 90% (27/30) positively expressed estrogen receptor (ER) and progesterone receptor (PR), respectively, while there were 23.33% (7/30) and 10% (3/30) negatively expressed ER and PR, respectively. HER2/neu was found to be positively expressed in 13.33% (4/30) of cases and negatively expressed in 86.66% (26/30) of breast cancer patients (Table 1).

**Table 1:** Clinicopathological characterization of early-diagnosed breast cancer patients

Parameter	Percentage (%)
Age	39–62 years
Clinical tumor grade	Stage I 3.33% (1/30)
	Stage II 70% (21/30)
	Stage III 26.66% (8/30)
Pathological type	Invasive ductal carcinoma (IDC) 83.33% (25/30)
	Ductal carcinoma in situ (DCIS) 10% (3/30)
	Multifocal carcinoma 6.66% (2/30)
Estrogen receptor (ER) status	Positive 76.66% (23/30)
	Negative 23.33% (7/30**)**
progesterone receptor (PR) status	Positive 90% (27/30)
	Negative 10% (3/30)
HER2/neu status	Positive 13.33% (4/30)
	Negative 86.66% (26/30)

### Expression levels of the circulating microRNAs among the investigated groups

**Fig. 1:**
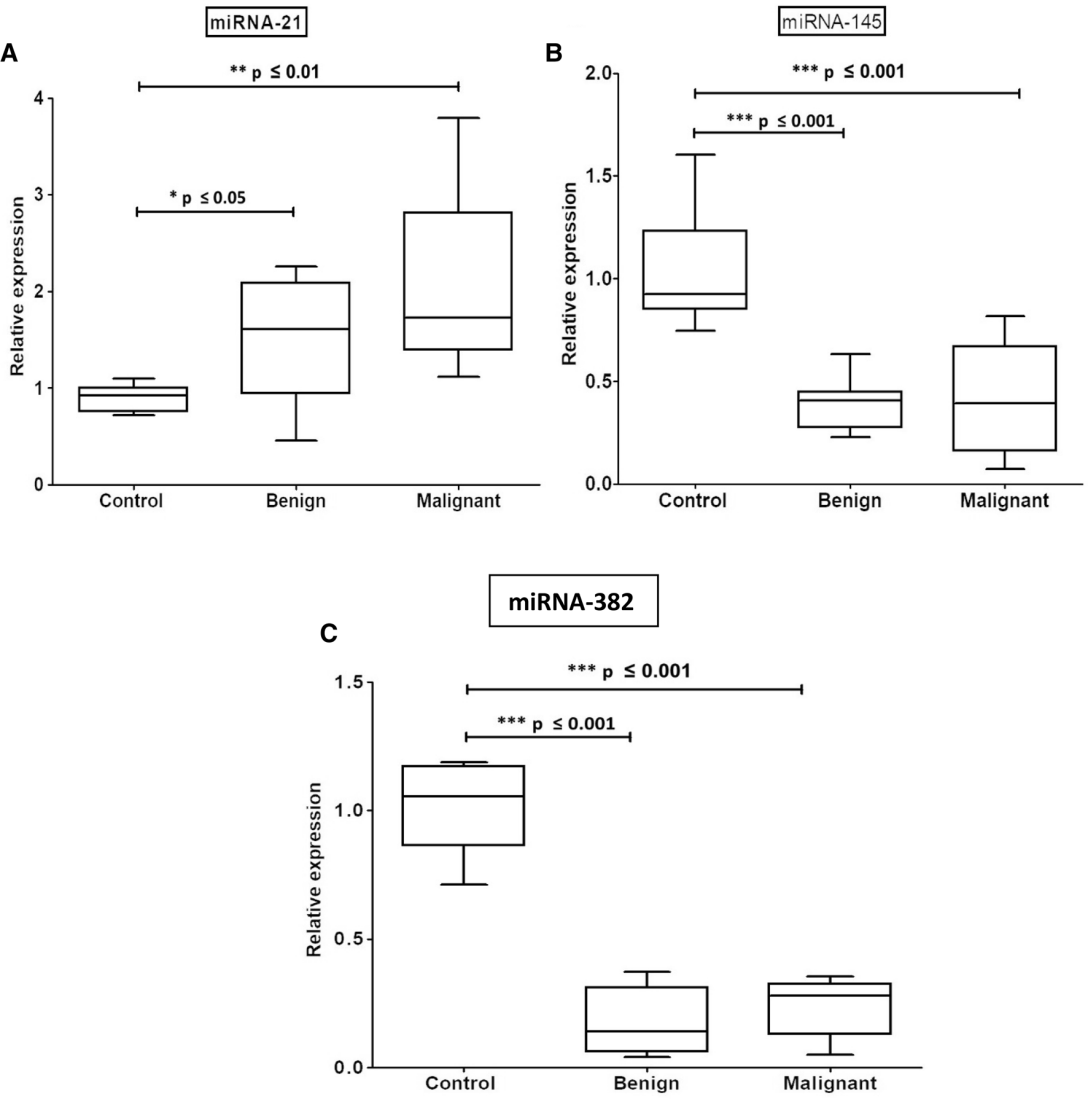
Whisker boxplot displays the relative expression patterns of serum miRNA-21 (**A**), -145 (**B**), and -382 (**C**) among the investigated study groups. Significant differences between the compared groups are also shown

As shown in Table 2 and Fig. 1, the median levels of the detected miRNAs −145, and −382 were recorded to be highly significantly decreased (*P* ≤ 0.001) in both benign and malignant groups as compared to control individuals. On the other hand, our data revealed a significant increase in the expression level of miRNA-21 in patients with breast lesions (benign) (*P* ≤ 0.05) and a highly significant increase in patients with breast cancer (*P* ≤ 0.01).

**Table 2:** The mean of the fold change of microRNA expression levels

MicroRNA	Control	Benign	Malignant
miRNA-145	1.032 ± 0.1241	0.3907 ± 0.05218^a^ (*P* ≤ 0.001)	0.4202 ± 0.0746^a^ (*P* ≤ 0.001)
miRNA-382	1.014 ± 0.06464	0.1731 ± 0.04228^a^ (*P* ≤ 0.001)	0.2244 ± 0.0302^a^ (*P* ≤ 0.001)
miRNA-21	0.9017 ± 0.05714	1.594 ± 0.2169^a^ (*P* ≤ 0.05)	2.103 ± 0.239^a^ (*P* ≤ 0.01)

^a^Significant as compared to control patients

### Estimation of glutamic acid concentration by LC/MS/MS

**Fig. 2:**
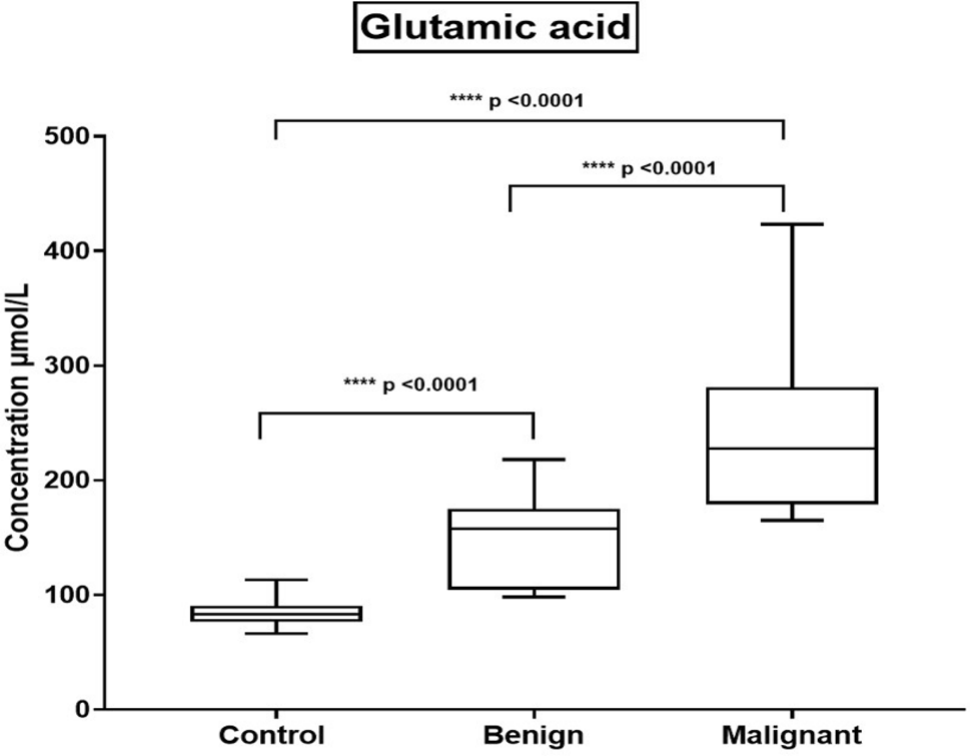
Whisker boxplot illustrates the mean values of the glutamic acid concentrations among the investigated study groups. Significant differences between the compared groups are also shown

Using the dried blood spot (DBS)-based mass spectrometry metabolomics analysis technique, breast tumor metabolite markers, amino acids and acylcarnitine profiles were screened (attached as Supplementary file). In light of this, a DBS-based metabolomic study was performed by using direct LC/MS/MS analysis of BC and the control samples in this study focusing on the detection of glutamic acid concentrations. As shown in Table 3 and Fig. 2, there was a highly significant difference between the benign and control groups. Furthermore, similar statistical significance was noticed between malignant and both benign and control groups.

**Table 3:** Detected mean values of glutamic acid concentrations for each studied group

Subjects	Mean (µmol/L) ± SEM
Control	84.05 ± 2.79
Benign	146.6 ± 7.39^**a^
Malignant	241.9 ± 13.52^**a,b^

**P ≤ 0.01

^a^As compared to the control group

^b^as compared to the benign group

### Serum concentration levels of HER2/neu

The evaluated mean concentration of HER2/neu in control healthy candidates was 6.1 ± 0.455 µg/L, while in patients with breast lesions (benign) and malignancy, it was calculated to be 8.4 ± 1.39 and 9.6 ± 1.8 µg/L, respectively. This slightly increase of HER2/neu in sera of malignant patients might be due to the presence of only four enrolled patients (4/30, 13.33%) who were recorded to be positively expressed HER2/neu in their provided medical reports.

### Pearson correlation coefficient (r) between all investigated parameters in all studied groups

**Fig. 3:**
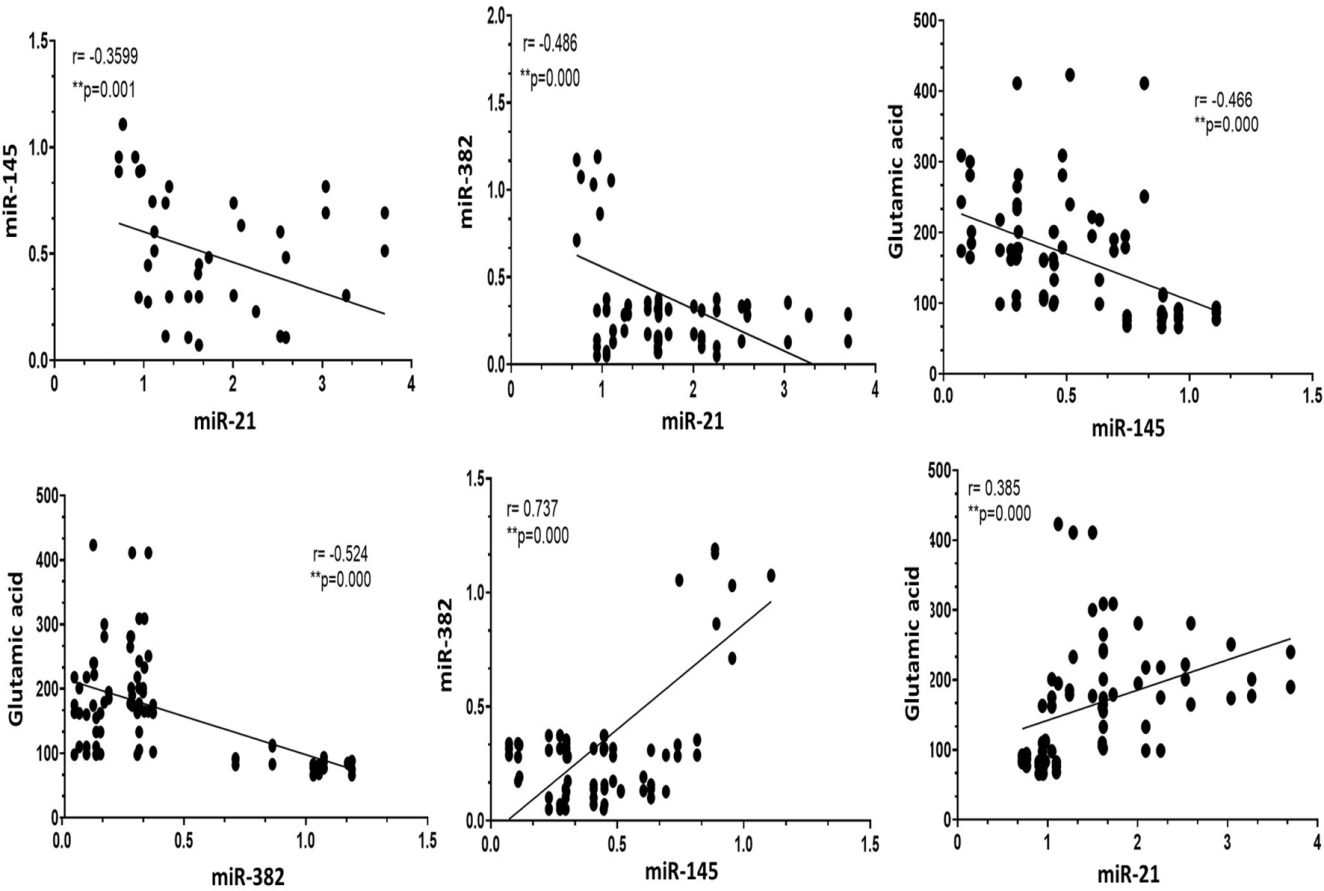
Pearson correlation coefficient (*r*) between the measured parameters in the different study groups

As indicated in Table 4 and Fig. 3, our data revealed a strong positive correlation between the expression pattern of miR-145 and miR-382 (*r* = 0.737) and a mild positive correlation between the expression of miR-21 and glutamic acid (*r* = 0.385). On the other hand, our data analysis revealed a negative correlation between the expression of miR-21with both miR-145 (*r* = −0.359) and miR-382 (*r* = −0.486). Also, a negative correlation was recorded between glutamic acid with miR-145 (*r* = −0.466) and miR-382 (*r* = −0.524) (*P* < 0.0001). Interestingly no significant correlations were detected between HER2/neu expression and the rest of the parameters.

**Table 4:** Pearson correlation coefficient (*r*) between the measured parameters in the different study groups

	miRNA-21	miRNA-145	miRNA-382	Glutamic acid	HER2/neu
miRNA-21	1	−0.359^**^	-0.486^****^	0.385^***^	0.154
miRNA-145	−0.359^**^	1	0.737^****^	−0.466^****^	−0.06
miRNA-382	−0.486^****^	0.737^****^	1	−0.524^****^	−0.125
Glutamic acid	0.385^**^	−0.466^****^	−0.524^****^	1	0.023
HER2/neu	0.154	−0.06	−0.125	0.023	1

^**^Pearson correlation (*r*) with statistically significant consideration at *P* ≤ 0.05 (*), *P* ≤ 0.01 (**), *P* ≤ 0.001 (***), and **** *P* < 0.0001 (2-tailed)

### Receiver operating characteristic (ROC) curve analysis

**Fig. 4:**
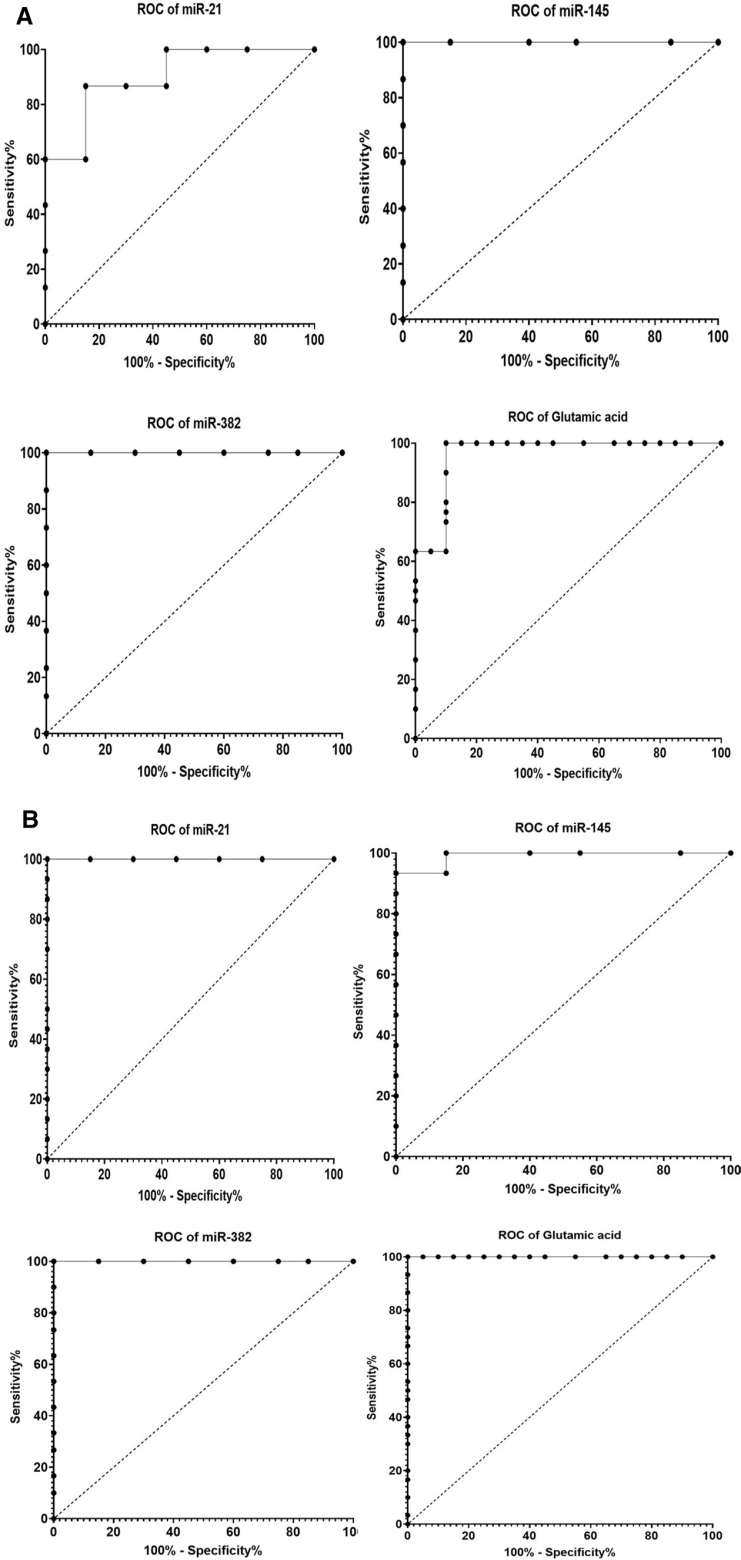

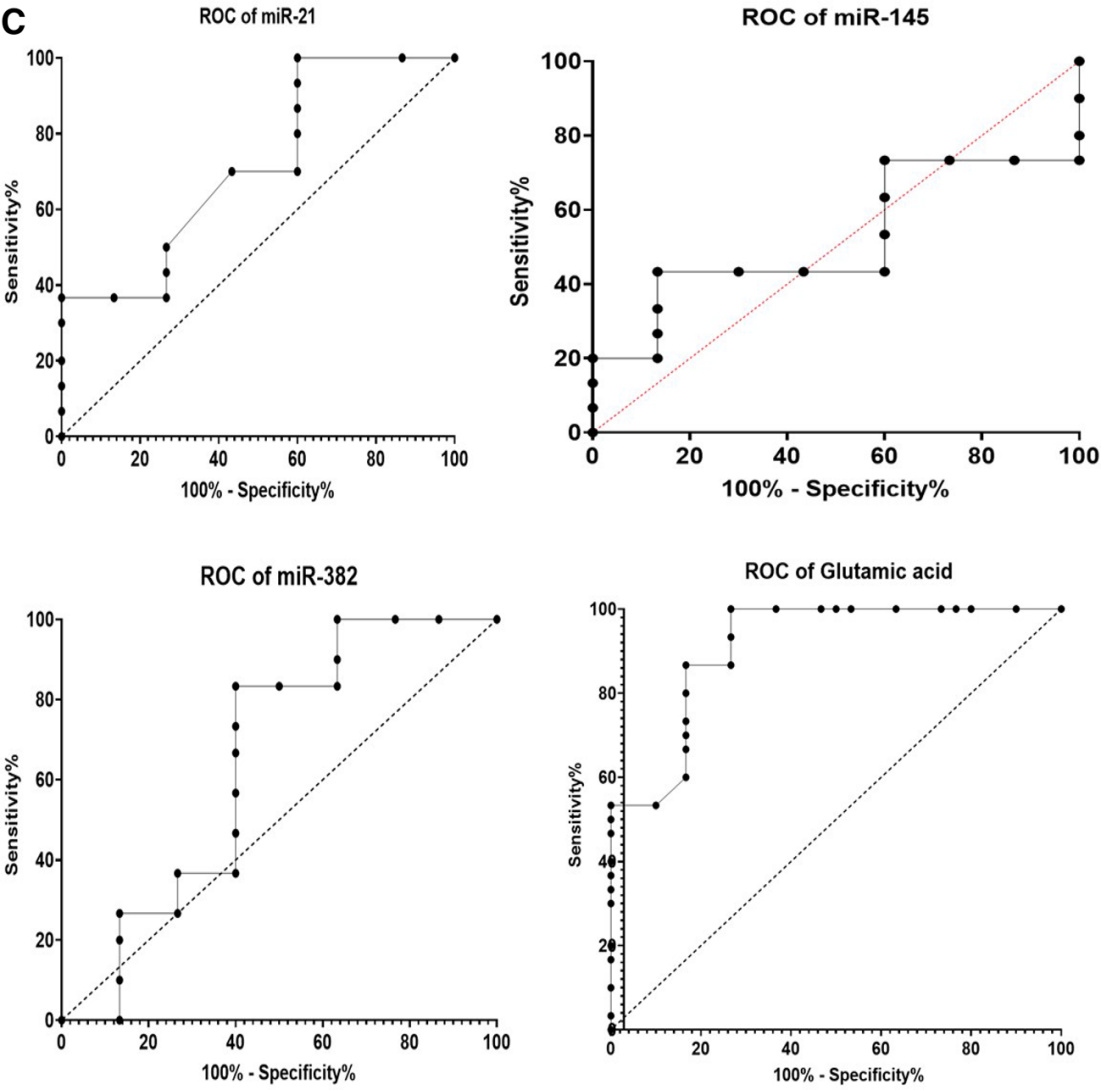
**A** Receiver operating characteristic (ROC) analysis of the measured parameters individually in benign samples, as compared to the control. **B** Receiver operating characteristic (ROC) analysis of the measured parameters individually in malignant patient samples, as compared to healthy subjects. **C** Receiver operating characteristic (ROC) analysis of measured parameters individually in malignant patient samples, as compared to benign cases

The most prominent approaches for evaluating the diagnostic accuracy of biomarkers are through assessing the area under the curve (AUC), in addition to sensitivity and specificity percentages as determined from ROC curves analysis. In the present study, we performed ROC analysis to each measured biomarker for data collected from control and benign samples (Table 5, Fig. 4A). The highest AUC, sensitivity, and specificity values were reported for circulating miRNA-145 and miRNA-382 (AUC = 1, sensitivity 100%, and specificity 100%). Moreover, the ROC curve analysis was carried out to compare between malignant and control subjects (Table 6, Fig. 4B). We observed highly significant AUC, sensitivity, and specificity values for all detected circulating parameters. Interestingly, when we conducted ROC curve analysis on results collected from benign and malignant samples (Table 7, Fig. 4C), the AUC, sensitivity, and specificity values were recorded to be lower. However, glutamic acid showed a reasonable AUC value, with moderate sensitivity % and specificity% (AUC = 0.91, sensitivity% = 86.67, specificity% = 83.33). In our analysis, we did not include the HER2 data, since only four subjects were HER2 positive in our study.

**Table 5:** Receiver operating characteristic (ROC) analysis of all measured parameters in the benign samples, as compared to control subjects

	AUC	95% confidence interval (CI)	*P* value	Cutoff value	Sensitivity %	Specificity %
miR-21	0.9	0.8166–0.9834	< 0.0001	> 1.011	86.67	85.00
miR-145	1	1.000–1.000	< 0.0001	< 0.6893	100.0	100.0
miR-382	1	1.000–1.000	< 0.0001	< 0.5425	100.0	100.0
Glutamic acid	0.9633	0.9108–1.000	< 0.0001	> 96.00	100.0	90.00

**Table 6:** Receiver operating characteristic (ROC) analysis of all measured parameters in malignant patients, as compared to healthy control samples

	AUC	95% Confidence interval (CI)	*P* value	Cutoff value	Sensitivity %	Specificity %
miR-21	1.00	1.000–1.000	< 0.0001	> 1.108	100	100
miR-145	0.99	0.9716–1.000	< 0.0001	< 0.8512	100	85
miR-382	1.00	1.000–1.000	< 0.0001	< 0.5329	100	100
Glutamic acid	1.00	1.000–1.000	< 0.0001	> 139.0	100	100

**Table 7:** Receiver operating characteristic (ROC) analysis of all measured parameters in malignant patient samples, as a comparison with benign cases

	AUC	95% Confidence interval (CI)	*P* value	Cutoff value	Sensitivity %	Specificity %
miR-21	0.7144	0.5849–0.8440	0.0043	> 1.612	70	56.67
miR-145	0.5222	0.3671–0.6774	0.7675	< 0.2971	73.33	40
miR-382	0.6456	0.4984–0.7927	0.0528	< 0.1653	83.33	60.00
Glutamic acid	0.9111	0.8397–0.9825	< 0.0001	> 176.0	86.67	83.33

## Discussion

Breast cancer represents the most commonly diagnosed cancer and the fifth cause of cancer related-death all over the world, with an estimated 2.3 million cases (24.5% of all cancer cases) and 685,000 deaths (15.5% of cancer deaths) in 2020. Well-diagnosed cases are predicted to reach about 4.4 million in 2070. The incidence and mortality of BC vary among countries (Lei et al. 2021). Although various treatment options for BC are available, its effective management is still difficult because of the deficiency of sensitive and specific biomarkers for early detection and disease monitoring. Accumulating evidence in the last years has highlighted the prospective use of peripheral blood circulating miRNAs in BC diagnosis, prognosis, and monitoring the response to therapeutic agents. Because of their structural stability and ease of isolation procedure, miRNAs are increasingly suggested as promising non-invasive biomarkers for BC early diagnosis (El-Daly et al. 2022; Escuin et al. 2021; Jang et al. 2021).

In the present study, we observed a highly significant decrease (*P* ≤ 0.001) in the detected expression level of miRNA-145 in both benign and malignant cases compared to controls. Additionally, the AUC value for miRNA-145 was estimated to equal 0.99, suggesting the potential use of miRNA-145 for early diagnosis to discriminate between breast tumor patients and normal controls.

Ng and the co-workers were the first to demonstrate the reduction of miRNA-145 level in the plasma of BC patients, but according to their result and similar to our finding, the aberrant expression of miR-145 was not significant enough to differentiate between benign and invasive breast cancer cases (Ng et al. 2013).

Our finding was also comparable with the study by Ibrahim et al. 2020, which reported that the plasma expression level of miRNA-145 (AUC = 0.70) was recorded to be significantly decreased (*P* < 0.01) at the initial BC diagnosis, as compared to healthy individuals. Also, Iorioet al. (2005) identified miRNA-145 to be down-regulated in breast cancer tissues as compared to normal tissues (Nakhaie et al. 2020; Tsai et al. 2018; Quan et al. 2018). This detected reduction in the expression level of miRNA-145 in BC cases was suggested to be as a result of the methylation of its promoter (Liu et al. 2017). According to the above studies, miR-145 functions by inhibiting proliferation, angiogenesis, and metastasis.

In contrast to our results, the tumor suppressor miRNA-145 was found to be significantly higher in the serum samples of BC patients from the Mexican population (p < 0.001), with AUC value of 0.9777 (Mar-Aguilar et al. 2013). The same pattern of elevation in the expression of circulating miRNA-145-5p in BC patients was also reported for HER-2-positive Kazakh patients (Ashirbekov et al. 2020). Ashirbekov et al. suggested that the measured expression level of miRNA-145 in serum/plasma/tissue of BC patients might vary according to the ethnicity of the studied population. In Lebanese patients, Itani et al. (2021) reported high expression of miRNA-145 in plasma of BC patients as compared to healthy subjects. Also, Gonzalez-Villasana et al. (2019) concluded that the levels of miRNA-145 packed in circulating exosomes were similar to those in healthy individuals with an increased concentration of exosomes isolated from BC sera than those of healthy controls, which reflects their role in BC progression.

Another miRNA, whose pattern of expression we evaluated is miRNA-382. Our results revealed that the expression level of miRNA-382 was significantly decreased (*P* ≤ 0.001) in subjects with benign or malignant tumors as compared to normal control. The AUC value was estimated to equal 1.00, so miRNA-382 could be a good diagnostic biomarker. In addition, our investigation recorded a strong significant positive correlation (*r* = 0.737) between miRNA-145 and miRNA-382. Our results agree with those of Tao and Wu (2016), who found that the expression level of miRNA-382 was significantly lower in tissue samples of Chinese BC patients compared with that in normal tissue. Moreover, a study performed by Li et al. (2013) showed that miR-382-5p expression was significantly decreased in tissues of patients with ductal carcinoma in situ (DCIS).

On the contrary, miRNA-382-3p was observed to be significantly up-regulated in the serum of breast cancer patients with AUC ranging from 0.740 to 0.9666, as compared to non-cancerous control (Zhang et al. 2019; Fu et al. 2016; Mar-Aguilar et al. 2013). The study by Gonzalez-Villasana et al. (2019) stated that miRNA-382 was detected in both exosomes isolated from sera of BC patients and healthy individuals, but with more elevated concentrations in BC cases. Generally, the oncogenic or tumor suppressive functions of miRNA-382 would be predicted depending on the targeting genes of miRNA-382 (Zhang et al. 2019; Lv et al. 2019; Feng et al. 2017).

Oncomir-21 in the current research was quantified by qRT-PCR and evaluated to be expressed in BC patients’ sera with significantly higher levels (*P* ≤ 0.01) than in benign and healthy women. The AUC was calculated to be 1.00 for miRNA-21 individually in BC patients as a comparison with control subjects. The underlying explanation for such elevation of miRNA-21 levels in BC could be related to its location on the region of chromosome 17q23.2 that is frequently amplified in breast tumors, or related to the hypomethylation of CpG island region that is located upstream of the mature miRNA-21 sequence (Elzoghby et al. 2019). Our data synchronize with the findings of Elzoghby et al. (2019), Swellam et al. (2018), Motawi et al. (2016), and Toraih et al. (2015), who showed that early diagnostic miRNA-21 expression levels were mostly up-regulated in the serum of Egyptian BC patients, followed by benign then healthy subjects, with AUC equal to 0.861. Additional harmonized investigations were found to agree with our results in the sera of studied ethnically diverse candidates (Salim et al. 2020; Han et al. 2017; Zhang et al. 2016a, b; Wang et al. 2015; Si et al. 2013; Gao et al. 2013). Many studies are in accordance with our results in confirming the increased expression of miRNA-21 in breast cancer tissue compared to the corresponding adjacent normal one, and recognizing miRNA-21 as one of the most important diagnostic biomarkers implicated in early detection of breast malignancy, since its expression is gradually up-regulated with the severity of the disease (Amirfallahet al. 2021; Wu 2020; Soleimanpour et al. 2019;Yanwirasti et al. 2017; Zhang et al. 2016a, b; Wang et al. 2015; Qian et al. 2009; Yan et al. 2008). Supportively, expression of circulating miRNA-21 was reported to be significantly elevated in plasma of breast cancer patients, with good to fair AUC values, compared to healthy individuals, and these amplified levels of miRNA-21 expression was observed to be reduced after completion of surgery and chemotherapy (Ibrahim et al. 2020; Savitri et al. 2020; Anwar et al. 2019; Soleimanpouret al. 2019; Jurkovicova et al. 2017).

However, Mar-Aguilar et al. (2013) and Chan et al. (2013) found comparable levels of miRNA-21 expression in BC patients and controls, and miR-21 was not identified as the most important diagnostic marker.

Mass spectrometry (MS)-based platforms have become an essential approach for rapidly screening and qualifying many biomarkers for further development and validation of BC. However, there is a persisting need to search for reliable biomarkers that can detect BC constantly at an early stage (Ducret et al. 2019). As tumor cells have distinct metabolic pathways and metabolites that are similar to their counterparts, liquid chromatography/mass spectrometry (LC/MS/MS) was used in the current study to investigate the amino acid profiles in dried blood spots (DBS) of BC patients, women with benign lesions, and control group. Amino acids are the basic building blocks for almost all cell types and specific amino acids were reported to be changed in plasma of numerous cancer patients, including breast and lung (Cascino et a. 1995). In the context of cancer metabolomics, many amino acids have been demonstrated to provide valuable clues for studying pathogenesis and to act as potential indicators for diverse malignancies (Kimura et al. 2009).

Our LC/MS/MS results elucidated gradual elevated levels of glutamic acid concentration among the studied groups. The mean concentration values were 84.05, 146.6, and 241.9 µmol/L for control, benign, and malignant subjects, respectively. High statistical significance (*P* ≤ 0.001) was observed between malignant and non-cancerous groups. Besides, AUC of malignant group as compared to control was detected to be 1.00 with associated high sensitivity and specificity percentages (100%). This gradual elevation of glutamic acid concentration among the studied groups indicates this amino acid to be a recent diagnostic biomarker that can discriminate between BC and healthy subjects. Wang et al. (2018) confirmed our results by using HPLC–MS/MS to evaluate the concentrations of free amino acids in serum of BC, benign, and healthy control groups. They reported a highly significant increase (*P* ≤ 0.001) in the mean glutamic acid concentration in the serum of BC patients (287.95 µmol/L) as compared to the benign (206.61 µmol/L) and control population (158.79 µmol/L). Additionally, they suggested that BC patients with higher level of taurine, glutamic acid, and lower ethylmalonic acid were likely to be diagnosed with BC. Accumulation of glutamic acid in the body causes low production of glutamine, enhancing proliferation and progression of breast carcinoma, and maintaining the Krebs cycle which is the main source of energy in the cells (Pietkiewicz et al. 2021; El Ansari et al. 2018; Wang et al. 2018; Cala et al. 2018a, b; Coloff et al. 2016). Similarly, additional spectroscopy-based metabolomics investigations support our findings (Cala et al. 2018a, b; More et al. 2018; Torata et al. 2018).

In contrast to our results, recent mass spectrometry-based studies conducted in plasma, serum and urine of BC patients to discover novel circulating diagnostic biomarkers for early detection of breast malignancy stated that the level of glutamic acid metabolite was found to be decreased in BC individuals in comparison with healthy control. The down-regulation of glutamic acid could be related to the high demand of amino acids in tumor metabolism (Yuan et al. 2019; Eniu et al. 2019; Cala et al. 2018a, b).

Overexpression of human epidermal growth factor receptor 2 (HER2) was found to be associated with clinically aggressive breast cancers with an elevation of metastasis, recurrence, and drug resistance. Amplification of HER2 occurs in about 15–30% of breast malignancies (Iqbal and Iqbal 2014; Ferretti et al. 2007). The function of nuclear HER2 could be directed by STAT3, which recruits HER2 to activate the expression of miRNA-21 that in turn down-regulates the expression of the metastasis suppressor protein programmed cell death 4 (PDCD4) in breast carcinoma. A negative correlation was recognized between circulating miRNA-21 and overall survival in HER2-positive breast cancers treated with neoadjuvant chemotherapy and trastuzumab or lapatinib (Amirfallahet al. 2021; Feng and Tsao 2016; Özgün et al. 2013). As our research was conducted in newly early-diagnosed women with non-invasive BC, the expression of HER2 was found to be positive in only 13.33% (4/30) patients. Thus, it is not a surprise to perceive the mean concentration of HER2 in BC sera (9.6 µg/L) to be slightly, but significantly higher than that in non-cancerous groups.

Totally, although current standard early diagnostic tools for detection of breast cancer are available, there is an urgent need for new minimally invasive diagnostic approaches to improve the screening rate of breast cancers and overcome the limitations of the current mammogram screening. Blood represents the primary source for discovery of potential breast cancer diagnostic biomarkers, which include miRNA-145, miRNA-382, miRNA-21, glutamic acid, and HER2/neu. All of these biomarkers have a role in the promotion of proliferation and progression of breast malignancy. Therefore, measuring the expression levels of these set of circulating diagnostic biomarkers will be valuable for early prediction of breast cancer and differntiate malignant patients from healthy subjects.

## Supplementary Information

Below is the link to the electronic supplementary material.

Supplementary file1 (DOCX 210 KB)

## Data Availability

All data are available in the current research paper.

## References

[CR1] Amirfallah A, Knutsdottir H, Arason A, Hilmarsdottir B, Johannsson OT, Agnarsson BA et al (2021) Hsa-miR-21-3p associates with breastcancer patient survival and targets genes in tumorsuppressive pathways. PLoS One 16(11):e0260327. 10.1371/journal.pone.0260327 34797887 10.1371/journal.pone.0260327 PMC8604322

[CR2] Anwar SL, Sari DNI, Kartika AI, Fitria MS, Tanjung DS, Rakhmina D, Wardana T, Astuti I, Haryana SM, Aryandono T (2019) Upregulation of circulating MiR-21 expression as a potential biomarker for therapeutic monitoring and clinical outcome in breast cancer. Asian Pac J Cancer Prev 20(4):1223–1228. 10.31557/APJCP.2019.20.4.1223 31030498 10.31557/APJCP.2019.20.4.1223 PMC6948877

[CR3] Ashirbekov Y, Abaildayev A, Omarbayeva N, Botbayev D, Belkozhayev A, Askandirova A, Neupokoyeva A, Utegenova G, Sharipov K, Aitkhozhina N (2020) Combination of circulating miR-145-5p/miR-191-5p as biomarker for breast cancer detection. Peer J 16(8):e10494. 10.7717/peerj 10.7717/peerj.10494 PMC7749656 33362968

[CR5] Cala MP, Aldana J, Medina J, Sanchez J, Guio J, Wist J et al (2018a) Multiplatformplasma metabolic and lipid fingerprinting of breast cancer: a pilotcontrol-case study in Colombian Hispanic women. PLoS One 13:e0190958 29438405 10.1371/journal.pone.0190958 PMC5810980

[CR6] Cala M, Aldana J, Sanchez J, Guio J, Meesters RJW (2018b) Urinary metabolite andlipid alterations in Colombian Hispanic women with breast cancer: a pilotstudy. J Pharm Biomed Anal 152:234–241 29428809 10.1016/j.jpba.2018.02.009

[CR7] Cascino A, Muscaritoli M, Cangiano C et al (1995) Plasma amino acid imbalance in patients with lung and breast cancer. Anticancer Res 15(2):507–510 7763031

[CR8] Chan M, Liaw CS, Ji SM, Tan HH, Wong CY, Thike AA et al (2013) Identification of circulating microRNA signatures for breast cancer detection. Clin Cancer Res 19:4477–4487 23797906 10.1158/1078-0432.CCR-12-3401

[CR9] Coloff JL, Murphy JP, Braun CR, Harris IS, Shelton LM, Kami K et al (2016) Differential glutamate metabolism in proliferating and quiescent mammaryepithelial cells. Cell Metab 23:867–880 27133130 10.1016/j.cmet.2016.03.016

[CR10] Ding Y, Zhang C, Zhang J, Zhang N, Li T, Fang J, Zhang Y, Zuo F, Tao Z, Tang S, Zhu W, Chen H, Sun X (2017) miR-145 inhibits proliferation and migration of breast cancer cells by directly or indirectly regulating TGF-β1 expression. Int J Oncol 50(5):1701–1710 28393176 10.3892/ijo.2017.3945

[CR11] Dowling P, Henry M, Meleady P, Clarke C, Gately K, O’Byrne K et al (2015) Metabolomic and proteomic analysis of breast cancer patient samples suggeststhat glutamate and 12-HETE in combination with CA15-3 may be usefulbiomarkers reflecting tumour burden. Metabolomics 11:620–635

[CR12] Ducret A, James I, Wilson S, Feilke M, Tebbe A, Dybowski N et al (2019) Translation and evaluation of a pre-clinical 5-protein response prediction signature in a breast cancer phase Ib clinical trial. PLoS One 14(3):e0213892 30897176 10.1371/journal.pone.0213892 PMC6428264

[CR13] El Ansari R, McIntyre A, Craze ML, Ellis IO, Rakha EA, Green AR (2018) Alteredglutamine metabolism in breast cancer; subtype dependencies and alternativeadaptations. Histopathology 72:183–190 28771772 10.1111/his.13334

[CR14] El-Daly SM, Morsy SM, Medhat D et al (2019) The diagnostic efficacy of circulating miRNAs in monitoring the early development of colitis-induced colorectal cancer. J Cell Biochem 120:16668–16680. 10.1002/jcb.28925 31095784 10.1002/jcb.28925

[CR15] El-Daly SM, Bayraktar R, Anfossi S, Calin GA (2020) The Interplay between MicroRNAs and the components of the tumor microenvironment in B-cell malignancies. Int J Mol Sci 21(9):3387. 10.3390/ijms21093387 32403283 10.3390/ijms21093387 PMC7246984

[CR16] El-Daly SM, Gouhar SA, Abd Elmageed ZY (2022) Circulating microRNAs as reliable tumor biomarkers: opportunities and challenges facing clinical application. J Pharmacol Exp Ther. 10.1124/jpet.121.000896 35809898 10.1124/jpet.121.000896 PMC9827506

[CR17] Elzoghby DMA, Mahmoud NH, Aly HH, Matar M (2019) Circulating microRNA-21 as a promising marker for early detection of breast cancer and disease progression in Egyptian females. Med J Cairo Univ 87:2491–2500. 10.21608/mjcu.2019.54858

[CR18] Eniu DT, Romanciuc F, Moraru C, Goidescu I, Eniu D, Staicu A et al (2019) Thedecrease of some serum free amino acids can predict breast cancer diagnosisand progression. Scand J Clin Lab Investig 79:17–24 30880483 10.1080/00365513.2018.1542541

[CR19] Escuin D, López-Vilaró L, Mora J, Bell O, Moral A, Perez I, Arqueros C, Garcia-Valdecasas B, Ramón y Cajal T, Lerma E, Barnadas A (2021) Circulating microRNAs in early breast cancer patients and its association with lymph node metastases. Front Oncol. 10.3389/fonc.2021.627811 34513655 10.3389/fonc.2021.627811 PMC8428362

[CR20] Feng YH, Tsao CJ (2016) Emerging role of microRNA-21 in cancer. Biomed Rep 5(4):395–402. 10.3892/br.2016.747 27699004 10.3892/br.2016.747 PMC5038362

[CR21] Feng J, Qi B, Guo L, Chen LY, Wei XF, Liu YZ, Zhao BS (2017) miR-382 functions as a tumor suppressor against esophageal squamous cell carcinoma. World J Gastroenterol 23(23):4243–4251. 10.3748/wjg.v23.i23.4243 28694664 10.3748/wjg.v23.i23.4243 PMC5483498

[CR23] Ferretti G, Felici A, Papaldo P, Fabi A, Cognetti F (2007) HER2/neu role in breast cancer: from a prognostic foe to a predictive friend. Curr Opin Obstet Gynecol 19(1):56–62. 10.1097/gco.0b013e328012980a 17218853 10.1097/GCO.0b013e328012980a

[CR24] Fu L, Li Z, Zhu J, Wang P, Fan G, Dai Y, Zheng Z, Liu Y (2016) Serum expression levels of microRNA-382-3p, -598-3p, -1246 and -184 in breast cancer patients. Oncol Lett 12(1):269–274. 10.3892/ol.2016.4582 27347136 10.3892/ol.2016.4582 PMC4906595

[CR25] Gao J, Zhang Q, Xu J, Guo L, Li X (2013) Clinical significance of serum miR-21 in breast cancer compared with CA153 and CEA. Chin J Cancer Res 25(6):743–748. 10.3978/j.issn.1000-9604.2013.12.04 24385703 10.3978/j.issn.1000-9604.2013.12.04 PMC3872537

[CR26] Gonzalez-Villasana V, Rashed MH, Gonzalez-Cantú Y, Bayraktar R, Menchaca-Arredondo JL, Vazquez-Guillen JM, Rodriguez-Padilla C, Lopez-Berestein G, Resendez-Perez D (2019) Presence of Circulating miR-145, miR-155, and miR-382 in exosomes isolated from serum of breast cancer patients and healthy donors. Dis Mark 2019:6852917. 10.1155/2019/6852917 10.1155/2019/6852917 PMC6390256 30891102

[CR27] Han JG, Jiang YD, Zhang CH, Yang YM, Pang D, Song YN, Zhang GQ (2017) A novel panel of serum miR-21/miR-155/miR-365 as a potential diag-nostic biomarker for breast cancer. Ann Surg Treat Res 92(2):55–66 28203552 10.4174/astr.2017.92.2.55 PMC5309178

[CR28] Hu J, Guo H, Li H, Liu Y, Liu J, Chen L, Zhang J, Zhang N (2012) MiR-145 regulates epithelial to mesenchymal transition of breast cancer cells by targeting Oct4. PLoS One 7(9):e45965. 10.1371/journal.pone.0045965 23049906 10.1371/journal.pone.0045965 PMC3458807

[CR29] Ibrahim AM, Said MM, Hilal AM, Medhat AM, Abd Elsalam IM (2020) Candidate circulating microRNAs as potential diagnostic and predictive biomarkers for the monitoring of locally advanced breast cancer patients. Tumor Biol. 10.1177/1010428320963811 10.1177/1010428320963811 33028151

[CR30] Iorio MV, Ferracin M, Liu C-G, Veronese A, Spizzo R, Sabbioni S, Magri E, Pedriali M, Fabbri M, Campiglio M, Ménard S, Palazzo JP, Rosenberg A, Musiani P, Volinia S, Nenci I, Calin GA, Querzoli P, Negrini M, Croce CM (2005) MicroRNA gene expression deregulation in human breast cancer. Cancer Res 65(16):7065–7070. 10.1158/0008-5472.CAN-05-1783 16103053 10.1158/0008-5472.CAN-05-1783

[CR31] Iqbal N, Iqbal N (2014) Human epidermal growth factor receptor 2 (HER2) in cancers: overexpression and therapeutic implications. Mol Biol Int 2014:852748. 10.1155/2014/852748 25276427 10.1155/2014/852748 PMC4170925

[CR32] Itani MM, Nassar FJ, Tfayli AH, Talhouk RS, Chamandi GK, Itani ARS, Makoukji J, Boustany R-MN, Hou L, Zgheib NK, Nasr RR (2021) A signature of four circulating microRNAs as potential biomarkers for diagnosingEarly-stage breast cancer. Int J Mol Sci 22:6121. 10.3390/ijms22116121 34204158 10.3390/ijms22116121 PMC8200990

[CR33] Jang JY, Kim YS, Kang KN, Kim KH, Park YJ, Kim CW (2021) Multiple microRNAs as biomarkers for early breast cancer diagnosis. Mol Clin Oncol 14:31 33414912 10.3892/mco.2020.2193 PMC7783718

[CR34] Jurkovicova D, Smolkova B, Magyerkova M, Sestakova Z, Kajabova VH et al (2017) Down-regulation of traditional oncomiRs in plasma of breast cancer patients. Oncotarget 8(44):77369–77384 29100393 10.18632/oncotarget.20484 PMC5652785

[CR35] Kim S-J, Oh J-S, Shin J-Y, Lee K-D, Sung KW, Nam SJ, Chun K-H (2011) Development of microRNA-145 for therapeutic application in breast cancer. J Contr Release 155:427–434. 10.1016/j.jconrel.2011.06.026 10.1016/j.jconrel.2011.06.026 21723890

[CR36] Kimura T, Noguchi Y, Shikata N et al (2009) Plasma amino acid analysis for diagnosis and amino acid-based metabolic networks. Curr Opin Clin Nutr Metab Care 12(1):49–53 19057187 10.1097/MCO.0b013e3283169242

[CR37] Lange T, Stracke S, Rettig R et al (2017) Identification of miR-16 as an endogenous reference gene for the normalization of urinary exosomal miRNA expression data from CKD patients. PLoS One 12(8):e0183435. 10.1371/journal.pone.0183435.PMID:28859135;PMCID:PMC5578666 28859135 10.1371/journal.pone.0183435 PMC5578666

[CR38] Lei S, Zheng R, Zhang S, Wang S, Chen R, Sun K, Zeng H, Zhou J, Wei W (2021) Global patterns of breast cancer incidence and mortality: a population-based cancer registry data analysis from 2000 to 2020. Cancer Commun 41(11):1183–1194. 10.1002/cac2.12207 10.1002/cac2.12207 PMC8626596 34399040

[CR39] Li S, Meng H, Zhou F, Zhai L, Zhang L, Gu F, Fan Y, Lang R, Fu L, Gu L, Qi L (2013) MicroRNA-132 is frequently down-regulated in ductal carcinoma in situ (DCIS) of breast and acts as a tumor suppressor byinhibiting cell proliferation. Pathol Res Pract 209:179–183 23399321 10.1016/j.prp.2012.12.002

[CR40] Li J, Guan X, Fan Z, Ching L-M, Li Y, Wang X, Cao W-M, Liu D-X (2020) Non-invasive biomarkers for early detection of breast cancer. Cancers 12(10):2767. 10.3390/cancers12102767 32992445 10.3390/cancers12102767 PMC7601650

[CR41] Link F, Krohn K, Schumann J (2019) Identifcation of stably expressed housekeeping miRNAs in endothelial cells and macrophages in an infammatory setting. Sci Rep 9:12786. 10.1038/s41598-019-49241-7 31484960 10.1038/s41598-019-49241-7 PMC6726651

[CR42] Liu SY, Li XY, Chen WQ, Hu H, Luo B, Shi YX, Wu TW, Li Y, Kong QZ, Lu HD, Lu ZX (2017) Demethylation of the MIR145 promoter suppresses migration and invasion in breast cancer. Oncotarget 8(37):61731–61741 28977900 10.18632/oncotarget.18686 PMC5617460

[CR43] Lv S, Huang Z, Liu J, Fan Z (2019) Effect of miR-382 on triple negative breast cancer cell line 4T1 by targeting PGC-1α. RNA Disease 6:e449. 10.14800/rd.449

[CR44] Lv P, Zhang Z, Hou L, Zhang Y, Lu L, Wang C, Shi F (2020) Meta-analysis of the clinicopathological significance of miRNA-145 in breast cancer. Biosci Rep. 10.1042/BSR20193974 10.1042/BSR20193974 PMC7502658 32869851

[CR45] Mar-Aguilar F, Mendoza-Ramírez JA, Malagón-Santiago I, Espino-Silva PK, Santuario-Facio SK, Ruiz-Flores P, Rodríguez-Padilla C, Reséndez-Pérez D (2013) Serum circulating microRNA profiling for identification of potential breast cancer biomarkers. Dis Markers 34(3):163–169. 10.3233/DMA-120957 23334650 10.3233/DMA-120957 PMC3810231

[CR46] Marrugo-Ramírez J, Mir M, Samitier J (2018) Blood-based cancer biomarkers in liquid biopsy: a promising non-invasive alternative to tissue biopsy. Int J Mol Sci 19(10):2877. 10.3390/ijms19102877 30248975 10.3390/ijms19102877 PMC6213360

[CR47] More TH, RoyChoudhury S, Christie J, Taunk K, Mane A, Santra MK et al (2018) Metabolomic alterations in invasive ductal carcinoma of breast: a comprehensive metabolomic study using tissue and serum samples. Oncotarget 9:2678–2696 29416801 10.18632/oncotarget.23626 PMC5788669

[CR48] Motawi MK, Sadik N, Shaker OG, El Mas-Ry MR, Mohareb F (2016) Study of microRNAs-21/221 as potential breast cancer biomarkers in Egyptian women. Gene 590(2):210–219 26827795 10.1016/j.gene.2016.01.042

[CR49] Nabih HK (2020) Crosstalk between NRF2 and dicer through metastasis regulating microRNAs; mir-34a, mir-200 family and mir-103/107 family. Arch Biochem Biophysics 686:108326. 10.1016/j.abb.2020.108326 10.1016/j.abb.2020.108326 32142889

[CR50] Nakhaie M, Makvandi M, Charostad J, Arabzadeh SAM, Motamedfar A, Kaydani GA (2020) Downregulation of miR-143/145 cluster in breast carcinoma specimens: putative role of DNA oncoviruses. Jundishapur J Microbiol 13(12):e110685. 10.5812/jjm.110685

[CR51] Ng EK, Li R, Shin VY, Jin HC, Leung CP, Ma ES, Pang R, Chua D, Chu KM, Law WL, Law SY, Poon RT, Kwong A (2013) Circulating microRNAs as specific biomarkers for breast cancer detection. PLoS One 8(1):e53141. 10.1371/journal.pone.0053141 23301032 10.1371/journal.pone.0053141 PMC3536802

[CR52] Orlandella FM, Auletta L, Greco A, Zannetti A, Salvatore G (2021) Preclinical imaging evaluation of miRNA’s delivery and effects in breast cancer mouse models: a systematic review. Cancers 13(23):6020. 10.3390/cancers13236020 34885130 10.3390/cancers13236020 PMC8656589

[CR53] Özgün A, Karagoz B, Bilgi O, Tuncel T, Baloglu H, Kandemir E (2013) MicroRNA-21 as an Indicator of Aggressive Phenotype in Breast Cancer. Onkologie 36:115–118. 10.1159/000348678 23485999 10.1159/000348678

[CR54] Pedroza-Torres A, Romero-Córdoba SL, Justo-Garrido M, Salido-Guadarrama I, Rodríguez-Bautista R, Montaño S, Muñiz-Mendoza R, Arriaga-Canon C, Fragoso-Ontiveros V, Álvarez-Gómez RM, Hernández G, Herrera LA (2019) MicroRNAs in tumor cell metabolism: roles and therapeutic opportunities. Front Oncol 9:1404. 10.3389/fonc.2019.01404 31921661 10.3389/fonc.2019.01404 PMC6917641

[CR55] Pietkiewicz D, Klupczynska-Gabryszak A, Plewa S, Misiura M, Horala A, Miltyk W, Nowak-Markwitz E, Kokot ZJ, Matysiak J (2021) Free amino acid alterations in patients with gynecological and breast cancer: a review. Pharmaceuticals 14(8):731. 10.3390/ph14080731 34451829 10.3390/ph14080731 PMC8400482

[CR56] Pinweha P, Rattanapornsompong K, Charoensawan V, Jitrapakdee S (2016) MicroRNAs and oncogenic transcriptional regulatory networks controlling metabolic reprogramming in cancers. Comput Struct Biotechnol J 14:223–233. 10.1016/j.csbj.2016.05.005 27358718 10.1016/j.csbj.2016.05.005 PMC4915959

[CR57] Qian B, Katsaros D, Lu L, Preti M, Durando A et al (2009) High miR-21 expression inbreast cancer associated with poor disease-free survival in early stage disease andhigh TGF-beta1. Breast Cancer Res Treat 117:131–140 18932017 10.1007/s10549-008-0219-7

[CR58] Quan Y, Huang X, Quan X (2018) Expression of miRNA-206 and miRNA-145 in breast cancer and correlation with prognosis. Oncol Lett 16(5):6638–6642. 10.3892/ol.2018.9440 30405803 10.3892/ol.2018.9440 PMC6202535

[CR59] Rakhmina D, Haryana SM, Aryandono T (2021) MiR-21 and mRNA PTEN expression levels and biomarker potential in breast cancer. Med Lab Technol J 7(1):46–58

[CR60] Salim B, Athira MV, Kandaswamy A, Vijayakumar M (2020) Investigation on staging ofbreast cancer using miR-21 as a biomarker in the serum. Int J Biomed Eng Technol 33(3):211–221

[CR61] Savitri M, Bintoro UY, Sedana MP, Muhammad MN, Romadhon PZ, Amrita PNA, Wijaya AY, Hendrata WM, Prayoga AA (2020) Circulating Plasma miRNA-21 as a superior biomarker compared to CA 15–3: assessment in healthy age matched subjects and different stage of breast cancer patients. Indones Biomed J 12(2):185–188

[CR62] Shukla S, Singh BK, Pathania OP, Jain M (2016) Evaluation of HER2/neu oncoprotein in serum & tissue samples of women with breast cancer. Indian J Med Res 143:S52–S58. 10.4103/0971-5916.191769 27748278 10.4103/0971-5916.191769 PMC5080929

[CR63] Si H, Sun X, Chen Y, Cao Y, Chen S, Wang H, Hu C (2013) Circulating microRNA-92a and microRNA-21 as novel minimally invasive biomarkers for primary breast cancer. J Cancer Res Clin Oncol 139(2):223–229 23052693 10.1007/s00432-012-1315-y PMC3549412

[CR64] Soleimanpour E, Babaei E, Hosseinpour-Feizi MA, Montazeri V (2019) Circulating miR-21 and miR-155 aspotential noninvasive biomarkers in Iranian Azeri patients with breast carcinoma. J Can Res Ther 15:1092–1097 10.4103/jcrt.JCRT_1227_16 31603116

[CR65] Swellam M, El Magdoub HM, Hassan NM, Hefny MM, Sobeih ME (2018) Potential diagnostic role of circulating MiRNAs in breast cancer: implications on clinicopathological characters. Clin Biochem 56:47–54 29679553 10.1016/j.clinbiochem.2018.04.013

[CR66] Tao Z, Wu J (2016) Lower expression of Mir-382 is associated with the development, progression and metastasis of breast cancer in the Chinese population. Int J Clin Exp Pathol 9(6):5912–5920

[CR67] Toraih A, Mohammed A, Farrag S, Wissa N, Hosny S (2015) Pilot study of serum MicroRNA-21 as a diagnostic and prognostic biomarker in Egyptian breast cancer patients. Mol Diagn Ther 19:179–190 26063582 10.1007/s40291-015-0143-6

[CR68] Torata N, Kubo M, Miura D, Ohuchida K, Mizuuchi Y, Fujimura Y et al (2018) Visualizing energy charge in breast carcinoma tissues by MALDImass-spectrometry imaging profiles of low-molecular-weight metabolites. Anticancer Res 38:4267–4272 29970560 10.21873/anticanres.12723

[CR69] Tsai HP, Huang S-F, Li C-F et al (2018) Differential microRNA expression in breast cancer with different onset age. PLoS One 13(1):19 10.1371/journal.pone.0191195 PMC5764434 29324832

[CR70] Underwood JJ, Quadri RS, Kalva SP, Shah H, Sanjeevaiah AR, Beg MS, Sutphin PD (2020) Liquid biopsy for cancer: review and implications for the radiologist. Radiology 294(1):5–17 31746691 10.1148/radiol.2019182584

[CR71] Wang S, Bian C, Yang Z, Bo Y, Li J, Zeng L, Zhou H, Zhao RC (2009) miR-145 inhibits breast cancer cell growth through RTKN. Int J Oncol 34:1461–1466 19360360

[CR72] Wang G, Wang L, Sun S, Wu J, Wang Q (2015) Quantitative measurement of serum microRNA-21 expression in relation to breast cancer metastasis in chinese females. Ann Lab Med 35:226–232. 10.3343/alm.2015.35.2.226 25729725 10.3343/alm.2015.35.2.226 PMC4330173

[CR73] Wang Q, Sun T, Cao Y, Gao P, Dong J, Fang Y, Fang Z, Sun X, Zhu Z (2016) A dried blood spot mass spectrometry metabolomic approach for rapid breast cancer detection. Onco Targets Ther 9:1389–1398. 10.2147/OTT.S95862 27042107 10.2147/OTT.S95862 PMC4795570

[CR74] Wang X, Zhao X, Chou J, Yu J, Yang T, Liu L, Zhang F (2018) Taurine, glutamic acid and ethylmalonic acid as important metabolites for detecting human breast cancer based on the targeted metabolomics. Cancer Biomark 23(2):255–268 30103303 10.3233/CBM-181500 PMC13078538

[CR75] Wang H, Tan Z, Hu H, Liu H, Wu T, Zheng C, Wang X, Luo Z, Wang J, Liu S, Lu Z, Tu J (2019) microRNA-21 promotes breast cancer proliferation and metastasis by targeting LZTFL1. BMC Cancer 19(1):738. 10.1186/s12885-019-5951-3 31351450 10.1186/s12885-019-5951-3 PMC6661096

[CR76] Wei M, Du Y, Wu X, Su Q, Zhu J, Zheng L, Lv G, Zhuang J (2020) A benign and malignant breast tumor classification method via efficiently combining texture and morphological features on ultrasound images. Comput Math Methods Med 2020:5894010. 10.1155/2020/5894010 33062038 10.1155/2020/5894010 PMC7547332

[CR77] WHO (2020). https://www.who.int/news-room/fact-sheets/detail/breast-cancer

[CR78] Witwer KW (2015) Circulating MicroRNA biomarker studies: pitfalls and potential solutions. Clin Chem 61(1):56–63. 10.1373/clinchem.2014.221341 25391989 10.1373/clinchem.2014.221341

[CR79] Wu X (2020) Expressions of miR-21 and miR-210 in breast cancer and their predictive values for prognosis. Iran J Public Health 49(1):21–29 32309220 PMC7152636

[CR80] Wu H-J, Chu P-Y (2022) Current and developing liquid biopsy techniques for breast cancer. Cancers 14(9):2052. 10.3390/cancers14092052 35565189 10.3390/cancers14092052 PMC9105073

[CR81] Xu WX, Liu Z, Deng F, Wang DD, Li XW, Tian T, Zhang J, Tang JH (2019) MiR-145 a potential biomarker of cancer migration and invasion. Am J Transl Res 11(11):6739–6753 31814885 PMC6895535

[CR82] Yan LX, Huang XF, Shao Q, Huang MY, Deng L, Wu QL, Zeng YX, Shao JY (2008) MicroRNA miR-21 overexpression in human breast cancer is associated with advanced clinical stage, lymph node metastasis and patient poor prognosis. RNA 14(11):2348–2360. 10.1261/rna.1034808 18812439 10.1261/rna.1034808 PMC2578865

[CR83] Yang L, Wang Y, Cai H, Wang S, Shen Y, Ke C (2020) Application of metabolomics in the diagnosis of breast cancer: a systematic review. J Cancer 11(9):2540–2551. 10.7150/jca.37604 32201524 10.7150/jca.37604 PMC7066003

[CR84] Yanwirasti, Harahap WA, Arisanty D (2017) Evaluation of MiR-21 and MiR-10b Expression of Human Breast Cancer in West Sumatera. Pak J Biol Sci 20:189–196 29023075 10.3923/pjbs.2017.189.196

[CR85] Ye D, Shen Z, Zhou S (2019) Function of microRNA-145 and mechanisms underlying its role in malignant tumor diagnosis and treatment. Cancer Manag Res 11:969–979. 10.2147/CMAR.S191696 30774425 10.2147/CMAR.S191696 PMC6349084

[CR86] Yuan BW, Schafferer S, Tang QQ, Scheffler M, Nees J, Heil J et al (2019) A plasmametabolite panel as biomarkers for early primary breast cancer detection. Int J Cancer 144:2833–2842 30426507 10.1002/ijc.31996

[CR87] Zeinali T, Mansoori B, Mohammadi A, Baradaran B (2019) Regulatory mechanisms of miR-145 expression and the importance of its function in cancer metastasis. Biomed Pharmacother 109:195–207. 10.1016/j.biopha.2018.10.037 30396077 10.1016/j.biopha.2018.10.037

[CR88] Zhang C, Liu K, Li T, Fang J, Ding Y, Sun L, Tu T, Jiang X, Du S, Hu J, Hu J et al (2016a) miR-21: a gene of dual regulation in breast cancer. Int J Oncol 48:161–172 26549725 10.3892/ijo.2015.3232

[CR89] Zhang J, Jiang C, Shi X, Yu H, Lin H, Peng Y (2016b) Diagnostic value of circulating miR-155, miR-21, and miR-10b as promising biomarkers in human breast cancer. Int J Clin Exp Pathol 9(10):10258–10265

[CR90] Zhang X, Zhao H, Zhang Y, Yang X, Zhang J, Yi M, Zhang C (2019) The MicroRNA-382-5p/MXD1 Axis Relates to Breast Cancer Progression and Promotes Cell Malignant Phenotypes. J Surg Res 246:442–449. 10.1016/j.jss.2019.09.018 31635836 10.1016/j.jss.2019.09.018

