# Supplementary figures and images for: The diagnostic significance of circulating miRNAs and metabolite profiling in early prediction of breast cancer in Egyptian women
Source: J Cancer Res Clin Oncol. 2022 Dec 2;149(8):5437–51. doi: 10.1007/s00432-022-04492-2 (PMC10349790; doi:10.1007/s00432-022-04492-2)

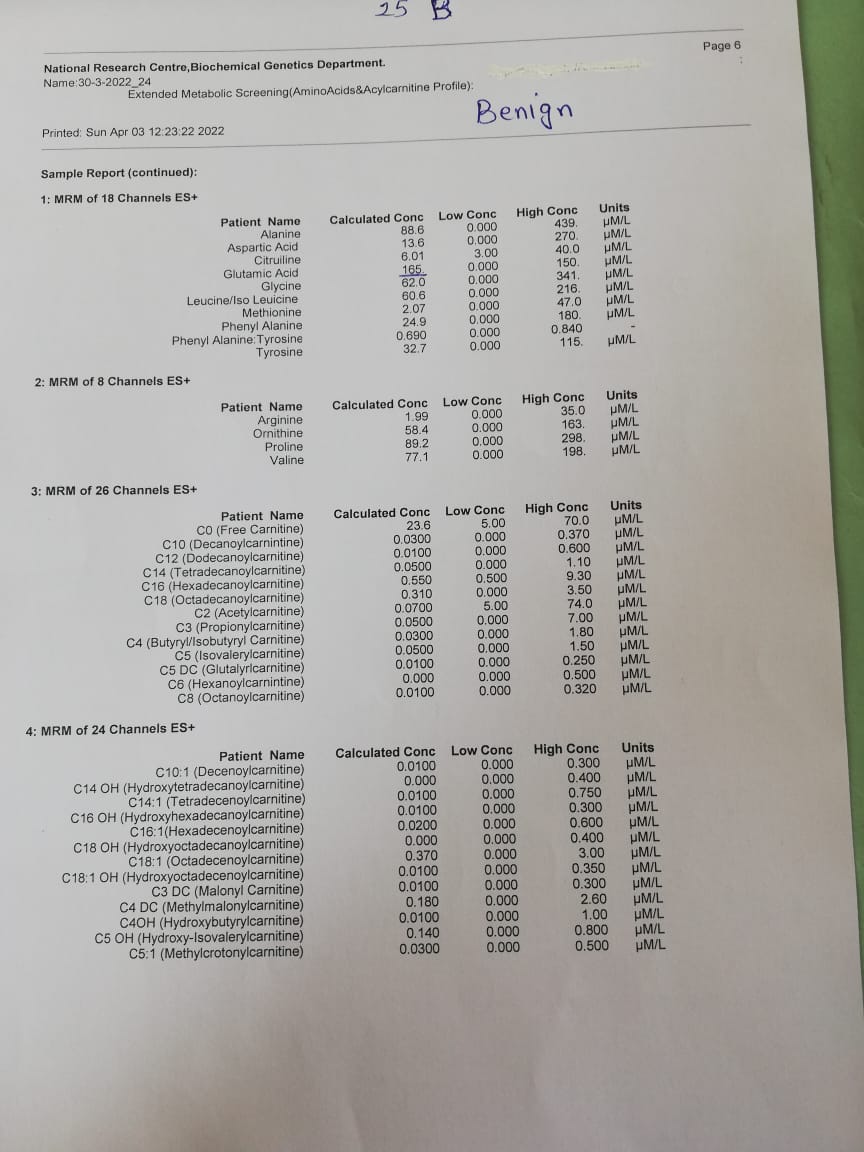


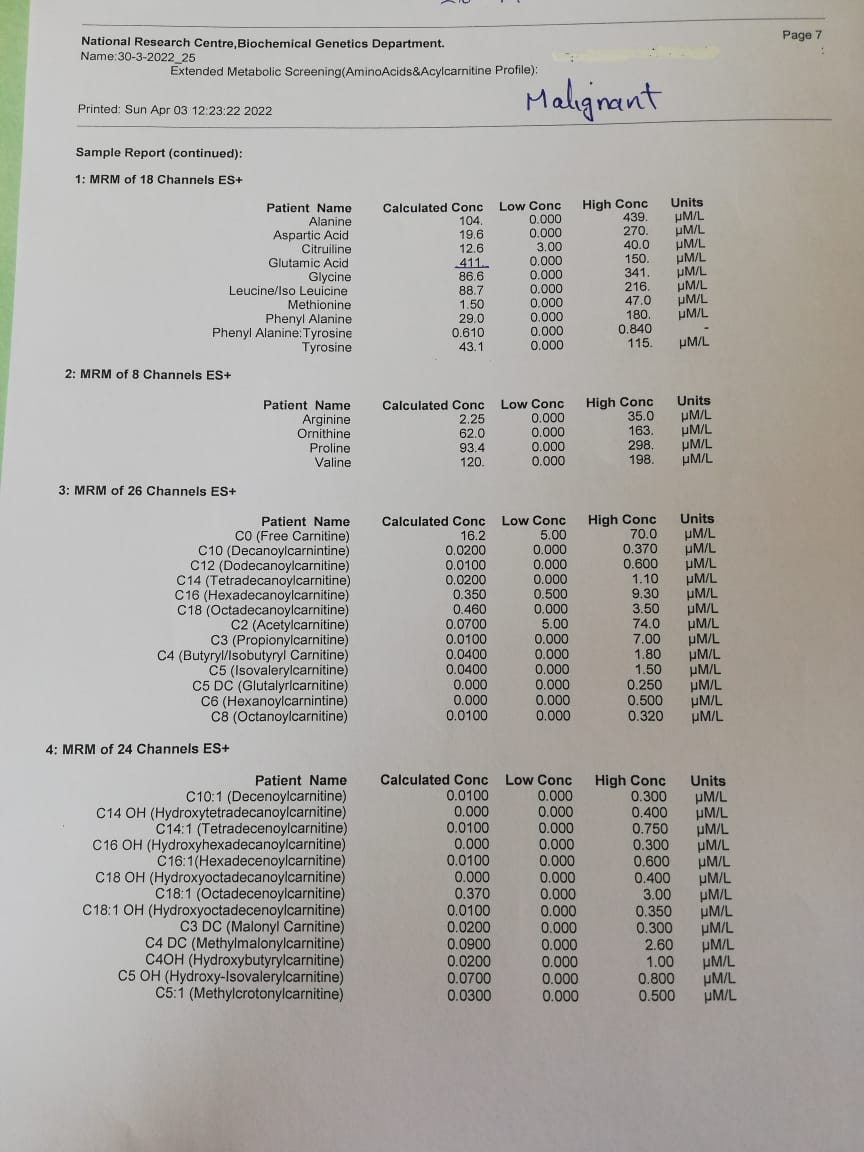

Supplement: Supplementary file 1 — Supplementary file1 (DOCX 210 KB) [file 432_2022_4492_MOESM1_ESM.docx]
